# A retrospective clinicopathological study on oral lichen planus and malignant transformation: Analysis of 518 cases

**DOI:** 10.4317/medoral.17778

**Published:** 2012-05-01

**Authors:** Zheng Y. Shen, Wei Liu, Lai K. Zhu, Jin Q. Feng, Guo Y. Tang, Zeng T. Zhou

**Affiliations:** 1 DDS. Shanghai Key Laboratory of Stomatology, Department of Oral Mucosal Diseases, Ninth People’s Hospital, Shanghai Jiao Tong University School of Medicine, Shanghai, China; 2 MD. Department of Dermatology, Ninth People’s Hospital, Shanghai Jiao Tong University School of Medicine, Shanghai, China; 3 DDS. School of Stomatology, Jilin University, Changchun, China

## Abstract

Objective: To investigate the epidemiological and clinical characteristics of a relatively large cohort of patients with oral lichen planus (OLP) from eastern China. Study design: A total of 518 patients with histologically confirmed OLP in a long-term follow-up period (6 months-21.5 years) were retrospectively reviewed in our clinic. Results: Of the 518 patients, 353 females and 165 males were identified. The average age at diagnosis was 46.3 years (range 9-81 years) with the buccal mucosa being the most common site (87.8%). At initial presentation, white lichen and red lichen was seen in 52.3% and 47.7% patients, respectively. Of these, 5 (0.96%) patients previously diagnosed clinically and histopathologically as OLP developed oral cancer. All of them were the females with no a history of smoking or alcohol use. Conclusions: Clinical features of eastern Chinese OLP patients were elucidated. Notably, approximately 1% of OLP developed into cancer, which provides further evidence of potentially malignant nature of OLP.

** Key words:**Oral lichen planus, clinical features, malignant transformation, oral cancer.

## Introduction

Oral lichen planus (OLP) is a relatively common inflammatory mucocutaneous disorder of uncertain etiology. It has a protracted clinical course despite various available treatments (1,2). The reported prevalence is 1% to 2% in the general population. There is a sex predilection with a female/male ratio of approximately 2:1, and the age of onset is generally between fourth and sixth decades of life. Buccal mucosa, usually bilateral, is the most affected site (3-15). Clinically, OLP maybe occur in 6 clinical variants as reticular, papular, plaque-like, erosive, atrophic and bullous. Histopathologically, OLP is characterized by dense subepithelial lymphohistiocytic infiltrate, increased numbers of intraepithelial lymphocytes, and degeneration of basal keratinocytes (16). Genital and cutaneous lichen planus are associated with approximately 20% and 15% of OLP, respectively (1,17). One of the most important complication concerning the progression and prognosis of OLP is the deve-lopment of oral squamous cell carcinoma (OSCC), with a frequency of malignant transformation of 0.4-5.3% (18), which led the WHO to classify OLP as a potentially malignant disorder (19). Thus, having OLP patients evaluated by a multidisciplinary group of health care providers is of great importance due to the concomitant lesion in extraoral sites involvement and oral cancer risk.

The demographic and clinical characteristics of OLP have been well described in several relatively large series of hundreds of cases from developed countries (3-12), whereas the large series from developing countries was scarce (13-15). Besides, there are no universally accepted specific clinically and histopathologically diagnostic criteria to date. Biopsy or surgery were not performed in all patients in several previous studies (5,10), while other disorders such as leukoplakia, erythroplakia, and discoid lupus erythematosus can present a similar clinical appearance. A major problem is the criteria of OLP malignant transformation due to differences in initial diagnosis, time of follow-up, and information on exposure to known oral carcinogens (6,7).

The objective of this retrospective study was to investigate the epidemiological and clinical characteristics of 518 OLP patients in eastern China (follow-up, 6 months-21.5 years). All the patients were diagnosed with OLP according to the clinical and histopathological criteria of the WHO.

## Material and Methods

All archived files of patients with the clinical and pathological diagnosis of OLP in the Department of Oral Mucosal Diseases, Ninth People’s Hospital, Shanghai Jiao Tong University School of Medicine from 1978 to 2009 were retrospectively reviewed. In our clinic, periodic follow-up examinations at intervals of every 6 months (or less) were recommended for patients diagnosed with OLP. All the study participants underwent biopsy. The biopsy was fixed in formalin, embedded in paraffin, and processed for routine histopathologic examination. Histopathologic diagnosis of OLP were made by oral pathologists on duty from the Department of Oral Pathology, Ninth People’s Hospital, Shanghai Jiao Tong University School of Medicine. As previously described (8), the WHO criteria (1978) for OLP (20) were used when examining the histopathology of the sections. The exclusion criteria were as follows.

I. Any patient with the clinical history and histopathologic changes of oral lichenoid lesion caused by an identifiable cause such as a hypersensitivity reaction to mechanical irritation; any other potentially malignant disorders: leukoplakia, erythroplakia, and discoid lupus erythematosus; and a bullous autoimmune disease: pemphigoid and pemphigus.

II. Any patient without the initial histopathologic diagnosis of OLP and development of OSCC during a follow-up period by biopsy or surgery.

III. Any patient with diagnosis of OLP concomitant OSCC at the first visit.

IV. Any patient with a follow-up period of less than 6 months after being diagnosed with OLP.

Based on these criteria, 518 patients with histologically confirmed OLP were selected to be retrospectively reviewed in this study. As described in previous studies (6,7), the clinical forms of OLP in this study were classified as white lichen in the presence of reticular, papular, or plaque-like lesions, and as red lichen in the presence of atrophic, erosive, or bullous lesions, independently of whether or not these coincide with white lichen at the periphery or in other sites. Information regarding age, gender, clinical form at the time of the initial diagnosis of OLP was all documented. History of smoking, alcohol use, systemic disease, and family history (i.e., in first-degree relatives) of OLP and oral cancer were also reviewed and analyzed. This study was approved by the institutional review board.

## Results

-Patient epidemiological characteristics

The epidemiological characteristics of OLP are presented in ( Table 1). There were 353 females and 165 males (ratio F: M = 2.1: 1). The mean age at diagnosis was 46.3 years, and the peak of age-frequency distribution was the fourth decade of life (32.8%). The buccal mucosa is the most common site (87.8%). Tongue and lip were affected in 295 (56.9%) and 77 (14.9%) patients, respectively. Multiple oral sites were affected in 250 (48.3%) patients. Buccal mucosa concomitant tongue were affected in 194 (37.5%) patients. Lesions only on the lip, gingiva, palate, and mouth floor were uncommon.

**Table 1:**
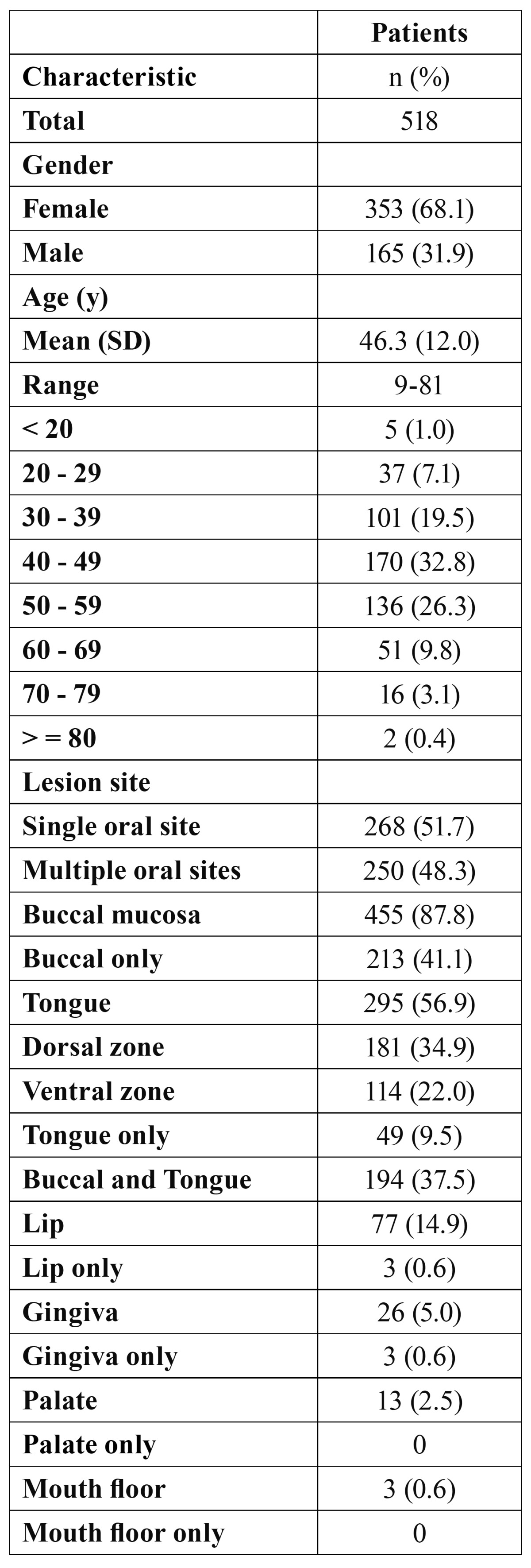
Patient epidemiological characteristics.

-Patient clinical characteristics

**Figure 1:**
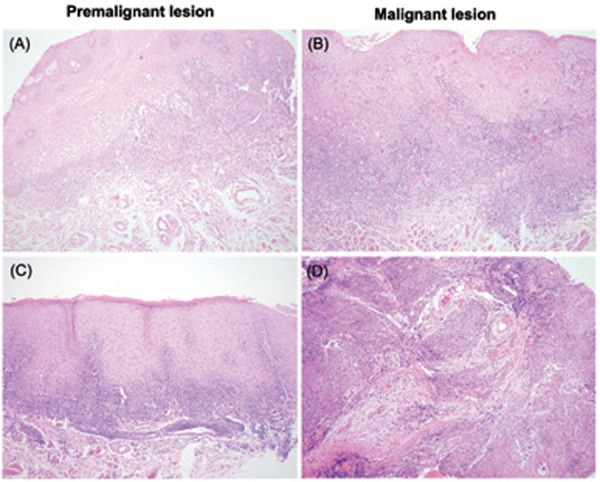
Histopathology of the premalignant and malignant OLP lesions. A,C) Premalignant histopathology. B,D) Malignant histopathology. Magnification,× 100.

Of the 518 patients, 5 (0.96%) patients previously diagnosed clinically and histologically as OLP developed oral cancer during the follow-up period ( Table 2). Representative histopathology of transformed OLP are shown in (Fig. 1), which illustrated the two cases (case 1 and case 2) in ( Table 2). All of them were the females with no a history of smoking or alcohol use. The clinical characteristics of OLP are listed in ( Table 3). At initial presentation, white lichen was seen in 271 (52.3%) patients, and red lichen was observed in 247 (47.7%) patients. History of smoking and alcohol use were reported in 43 (8.3%) and 41 (7.9%) cases, respectively. Family history of OLP and oral cancer were documented in 6 (1.2%) and 2 (0.4%) patients, respectively. The incidence of systemic diseases included hypertension (10.0%), Hepatitis B (6.9%), arthritis (1.7%), and diabetes mellitus (1.4%).

**Table 2:**
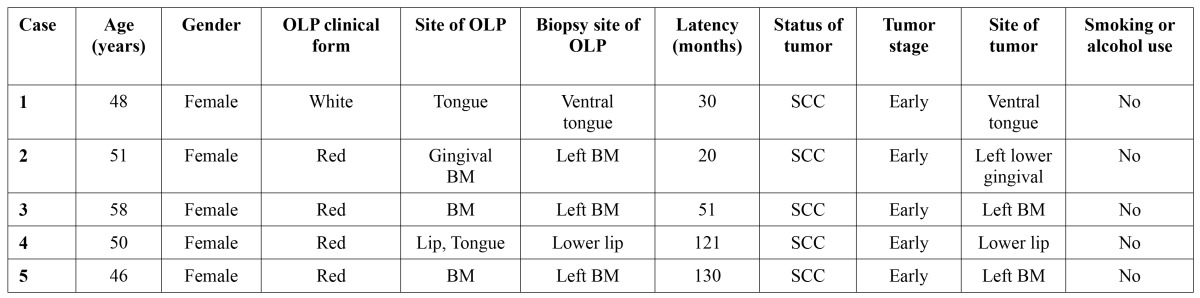
Characteristics of OLP patients with malignant transformation.

**Table 3:**
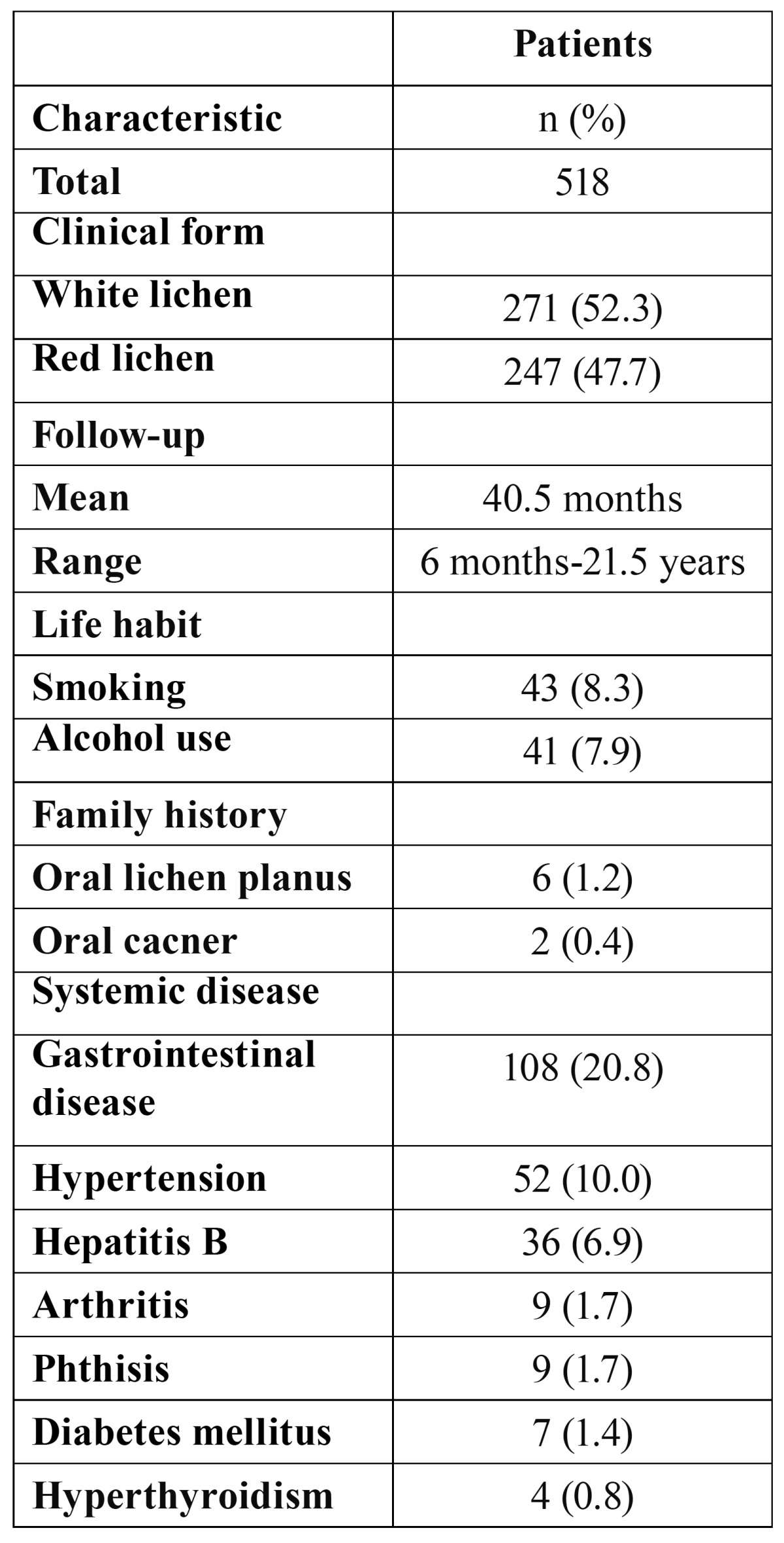
Patient clinical characteristics.

## Discussion

The present study attempts to elucidate the epidemiological and clinical characteristics of OLP patients in a relatively large cohort from eastern China. According to the clinical and histopathological criteria of the WHO, the results of this study reveal that OLP is typically middle-aged, sex predilection for female, and it usually affected the bilateral buccal mucosa and tongue. The frequency of OLP malignant transformation was 0.96% in this series. In this study, we excluded any patient with diagnosis of OLP concomitant OSCC at the first visit, and with a followed-up period of less than 6 months after the initial diagnosis of OLP. A short time interval between diagnosis of OLP and OSCC could lead to overestimation of the true incidence of OLP transformation and potentially suggest these two diseases were synchronous, in agreement with the criteria of the published literature (6-8).

We observed the women outnumbered the men (ratio F: M = 2.1: 1) in this study, in agreement with other reports (4,11-15). OLP is more prevalent in fourth decade of life in our study (mean age, 46.3 years), which is lower than the mean age reported in central China (50.4 years), UK (52.0 years), Spain (56.4 years), and Italy (56.7 years) (5,6,8,15). This was probably due to the ethnic population and geographic difference in our cohorts compared to previous reports. OLP in minor juveniles or children is uncommon and in our study it was observed in 5 patients, age ranging from 9 to 17 years, and 3 were with red lichen and 2 were with white lichen. To our knowledge, the 9-year-old child was the most youngest patient with histologically confirmed OLP. Although childhood OLP is few, early recognition is very important to make appropriate treatment and relieve symptoms of little children.

As previously mentioned, the lesions of OLP were typically bilateral and the buccal mucosa was the most common site of involvement, followed by the tongue (4-7,11-15). In a different way, we reported the lip was the third most common site, and buccal mucosa concomitant tongue were the most common multiple oral sites in our survey. Few lesions only located on gingiva, palate, and mouth floor, whereas these sites usually concomitant buccal mucosa or tongue were affected in multiple oral sites.

According to 2 categories of clinical form classified by Gandolfo et al. (6) and Carbone et al. (7), the prevalences of white lichen in their series were 59.7% and 58.9%, respectively. Likewise, the prevalence of white lichen in our series was 52.3% in the initial lesions. As reported by Eisen (4), most patients with OLP in our study show no increased prevalence of smoking and/or alcohol use, which do not seem to play a role in the pathogenesis of OLP. Besides, 6 of the present group of patients had family members with a history of OLP. Bermejo-Fenoll et al. (8) recently reported 5 families with 2 affected members, and 1 family with 3 affected members. These did not provide definitive insight into a genetic basis, whether OLP and development of OSCC has a strong genetic etiopathogenesis was not established by this study.

The incidence of the history of systemic diseases including hypertension (10.0%), arthritis (1.7%), diabetes mellitus (1.4%), hyperthyroidism (0.8%) was not higher than expected when compared with the incidence reported in the general population. Moreover, the incidence of these systemic diseases was lower than the previous reports (4,8,15,21). This indicates systemic diseases may not have a role on the pathogenesis of OLP. Although the correlation of OLP with diabetes mellitus has been suggested (22), the study by Xue et al. (15) and our present study did not support this observation in China.

A point to highlight was that we observed all of the 5 patients who developed OSCC were females with no a history of smoking or alcohol use, which was not in agreement with previous reports (6-8,15). Previous studies reported a part of patients with malignant transformation were the males, and smoker or alcohol user, which were not observed in our study. It is plausible to speculate that gender specific factors such as hormone replacement therapy, human papilloma virus infection maybe correlate with an increased risk of transformation in our area and population. Moreover, smoking and alcohol use were not observed in the present OLP patients with transformation. This may further demonstrate that OLP is of the true potentially malignant nature, independently of exposure to smoking and alcohol use of oral carcinogens.

In conclusion, the present investigation elucidated the epidemiological and clinical characteristics of patients with OLP and provided further evidence of potentially malignant nature of OLP in a relatively large cohort with long-term follow-up from eastern China.
